# Factors Influencing the Duration of Rehabilitation in Infants with Torticollis—A Pilot Study

**DOI:** 10.3390/medicina60010165

**Published:** 2024-01-16

**Authors:** Daniela Parau, Anamaria Butila Todoran, Rodica Balasa

**Affiliations:** 1 Doctoral School, ‘George Emil Palade’ University of Medicine, Pharmacy, Science, and Technology of Targu Mures, 540142 Targu Mures, Romania; 2 Department of Genetics, ‘George Emil Palade’ University of Medicine, Pharmacy, Science, and Technology of Targu Mures, 540142 Targu Mures, Romania; 3 Department of Neurology, ‘George Emil Palade’ University of Medicine, Pharmacy, Science, and Technology of Targu Mures, 540136 Targu Mures, Romania; rodica.balasa@umfst.ro

**Keywords:** torticollis, Vojta therapy, intervention, infant, rehabilitation

## Abstract

*Background and Objectives*: Torticollis is a common pediatric condition, with an incidence of 0.3–2.0%. Studies show that an adequate, tailored, and early treatment helps 90% to 95% of children recover before the first year of life and 97% of patients recover if treatment starts before the first six months. To identify the relationships between variables considered essential in the recovery process of infants with torticollis, we included factors such as the type of torticollis, age at onset of treatment, gender, birth weight, mode of delivery, fetal position in the uterus, the presence of craniofacial deformities, regions affected by postural asymmetries, and duration of the rehabilitation program. The hypothesis of the study is that early initiation of therapy can contribute to achieving favorable outcomes in the recovery process. *Material and Methods*: This retrospective cohort pilot study was conducted within a rehabilitation facility, spanning a duration of 1 year. The study involved a population of 41 children aged between 0 and 6 months. The rehabilitation program consisted of the application of Vojta therapy. Each session lasted 20 min, with a frequency of three times per week. *Results*: A total of 41% of those who started therapy in the first 3 months of life were fully recovered after 4–6 weeks of therapy. Of infants who started therapy at 5 and 6 months of age, 15% showed no improvement in measurements from 14 to 16 weeks of age, at which point the use of a cranial orthosis was recommended, and 23% experienced a plateau in measurements from 10 to 14 weeks, requiring the use of a cervical collar in conjunction with therapy. *Conclusions*: The findings from the study suggest that there may be a correlation between early initiation of therapy and favorable outcomes in the recovery process. The primary factors influencing the duration of recovery were identified as the presence of body asymmetries and the age at which therapy was initiated.

## 1. Introduction

Torticollis is a common pediatric condition, with an incidence of 0.3–2.0% [1,2] in healthy newborns [3], in which fibrosis and unilateral shortening of the sternocleidomastoid muscle (SCM) occurs leading to an asymmetric position of the head and neck [4,5]. It belongs to the category of non-paroxysmal cervical dystonia types [6]. With the SCM contraction, the range of motion at the neck level is limited (LOM) [7], causing a rotation of the face to the contralateral side and homolateral head tilt [8]. The etiology and pathogenesis of congenital muscular torticollis (CMT) are not fully understood [9], with pelvic presentation, cord circling, birth trauma, and vacuum or forceps applications being incriminated [6,10]. The etiology of acquired (postnatal) torticollis (AMT) can be environmental, induced by craniosynostosis (plagiocephaly), or induced by preferential positions, in case of cerebral palsy, Down syndrome, myelodysplasia, and dysfunction of the C1 occiput-cervical area [11]. The diagnosis of CMT is confirmed by ultrasound and clinical examination [12].

Torticollis can be associated with several deformities, which over time will affect the harmonious development of the infant and favor some vicious or compensatory postures both in infancy and adolescence [13,14]. Such deformities are represented by plagiocephaly, scoliosis, homolateral mandibular asymmetry, ear deformities on the affected side, pelvic asymmetry, coxo femoral luxation (it is estimated that 1 in 5 children with CMT have subluxation or hip dislocation), and leg deformities [7,15,16,17,18,19]. At present, there is no standardized therapeutic intervention, and in practice there are several approaches to treating CMT through the injection of botulinum toxin [20,21], physiotherapy [22], osteopathy [23,24,25], and surgery [26,27,28,29]. However, studies show that an adequate, tailored, and early treatment helps 90% to 95% of children recover before the first year of life and 97% [26] of patients recover if treatment starts before the first six months [26,30,31,32,33]. The main treatment remains physiotherapy, with invasive methods, such as surgery [34], used when therapy does not show any results or for cosmetic reasons [26,35,36,37].

In the present study, Vojta therapy is used as a kinesiological method of intervention for congenital muscular torticollis and acquired torticollis. Vaclav Vojta (1917–2000) developed a method of early diagnosis and treatment of neuro-developmental disorders in infants and came up with the therapeutic concept of releasing global motor complexes by stimulating appropriate areas on the patient’s body. Global motor complexes such as reflex locomotion—crawling and rotation—consist of all partial movement patterns, which are gradually used by a healthy infant in the process of postural and motor ontogenesis [38]. Providing the central nervous system with adequate external stimulation allows, using neural plasticity, the recreation of access to the human postural developmental program and the gradual replacement of pathological motor patterns with more regular ones [39]. The application of the Vojta technique improves automatic postural control and facilitates movement of the lower limbs, training, in particular, the autochthonous muscles of the spine, causing a synergistic involvement and collaboration of the muscle groups in the trunk and those around the key joints of the body [40,41].

To identify the relationships between variables considered essential in the recovery process of infants with torticollis, we included factors such as the type of torticollis, age at onset of treatment, gender, birth weight, mode of delivery, fetal position in the uterus, the presence of craniofacial deformities, regions affected by postural asymmetries, and duration of the rehabilitation program.

The present study was conducted to identify factors influencing the duration of therapy for infants with torticollis.

The hypothesis of the study is that early initiation of therapy can contribute to achieving favorable outcomes in the recovery process.

## 2. Material and Methods

This retrospective cohort pilot study was conducted within a rehabilitation facility named Therapy Kids, located in Târgu Mureș, Romania, spanning a duration of 1 year from 2022 to 2023. The study involved a population of 41 children aged between 0 and 6 months, selected from the patient pool of the rehabilitation facility, specifically chosen based on adherence to the predetermined inclusion and exclusion criteria outlined in the study protocol. One therapist conducted the evaluations, while the other applied the Vojta therapy. The study was conducted in accordance with the Declaration of Helsinki, and the study protocol was approved by the Scientific Ethics Committee of the University of Medicine, Pharmacy, Science, and Technology “George Emil Palade” of Targu Mures, under no. 962/03.06.2020. Written consent was obtained from the guardians of all patients prior to participation in the study.

Participants: The study group consisted of 41 infants aged 0–6 months, all diagnosed with torticollis. The diagnosis was established following a neurological evaluation, which revealed a clinical appearance consisting of a deviation of the head from the midline of the body and a limitation of range of movement (LOM) in terms of rotation and lateral tilt of the head. The diagnosis was subsequently completed by an ultrasonographic (USG) examination, a procedure used to measure the thickness of the SCM muscle [3,42,43].

Inclusion criteria: infants aged 0–6 months with confirmed congenital and acquired torticollis with craniofacial and body asymmetry, with a medical recommendation from the neurologist to initiate rehabilitation treatment, and born within 37–42 weeks. For all these children, written consent was obtained from their guardians.

Exclusion criteria: bony, neurogenic, ophthalmologic torticollis, post clavicle fracture, post cervical spine dislocation or subluxation, congenital musculoskeletal anomalies-hip dysplasia, premature infants, neurological disorders, genetic syndromes, and patients who have undergone SCM surgery. Participants who did not provide written consent to be involved in the study were also excluded.

Clinical evaluation by a physiotherapist with pediatric training consisted of measuring and recording, for each study participant, the degrees of rotation and lateral tilt of the head in the transversal and frontal planes using an arthrodial goniometer—an instrument recently recommended in the American Physical Therapy Association’s clinical practice guideline for the management of physiotherapy in torticollis [44,45]. During the measurement procedure, the infant was placed on the therapy table in a supine position and was kept in this position to prevent any body movements. The physiotherapist performed gentle rotational and lateral tilting movements of the infant’s head until encountering resistance. This moment was considered a limitation of movement, and the corresponding degrees were identified from the goniometer and recorded. Infant medical records were accessed through a rigorous process, involving proper authorization and adherence to data confidentiality norms. Relevant information for the study was extracted from patient medical records. Subsequently, an assessment sheet was created for each participant, recording gender, type of torticollis, age at the start of therapy, birth weight, mode of delivery, fetal position, presence of cranial deformities, facial and postural asymmetries, affected region, duration of the rehabilitation program, and the measurement of the rotation and lateral tilt of the head. Subsequently, the information obtained from the assessments was organized and structured using Excel software and databases by Microsoft 365.

**Figure 1:**
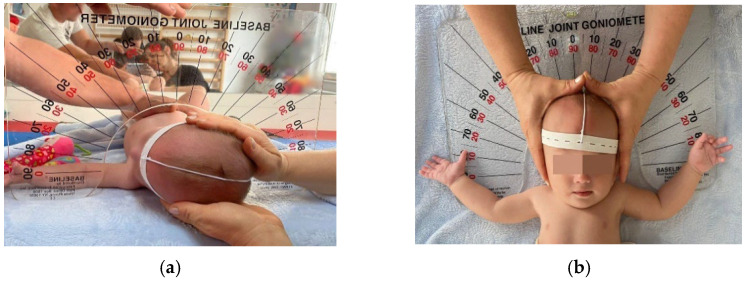
(**a**) PROM measurement of lateral rotation. (**b**) PROM measurement of lateral flexion.

The investigation included measurement of the passive range of motion (PROM) of lateral flexion (left/right) and rotation (left/right) using a equipment Baseline^®^ Arthrodial Protractor manufactured by Fabrication Enterprises White Plains, NY 10602 (USA), which has two opposing scales, 0–90° (left and right) in 5° increments, and the included spirit level ensures measurement accuracy, making it a reliable instrument for assessing infants (Figure 1a,b).

The calculation of cervical ROM (range of motion) deficits was performed as follows:

1. Lateral flexion—normal ROM is between 0° and 45°, but in the case study, the lateral tilt position between 5° and 45° on the affected side was considered a limitation of movement, and its normalization is reaching the zero-neutral point (0°).
2. Lateral rotation—normal ROM is 90°. In this study, intermediate values are defined as representing a restriction of movement. For example, when a PROM of 65° was recorded, a difference of 25° from normal values was identified as a deficit in lateral rotation that required intervention. Infants with an angle of 90° or more were classified as within normal values and treatment was considered optimal, and therapy was discontinued.

The focus of the remedial treatment was to achieve normal function of the SCM muscle, monitoring included initial measurements that were repeated at two-week intervals. The measurement results were noted and compared with the limitation norms in the specialized literature [46,47].

### 2.1. Rehabilitation Method

Vojta therapy is a method of treatment for infants younger than 6 months [6] with central nervous system (CNS) and musculoskeletal disorders, consisting of specific stimulation in certain areas (there are 10 zones distributed on the trunk, arms, and legs) [48] and positions (decubitus dorsal, decubitus ventral, and decubitus lateral). This stimulation causes the patient’s body to perform reflex movements so that the two movement complexes contained in the locomotion components—reflex crawling and reflex rolling—can be activated at the CNS level. The aim of the therapy is to activate and maintain correct physiological movement patterns, thus achieving a coordinated and rhythmic activation of the entire skeletal musculature. Vojta therapy can be used in a variety of movement-related conditions as well as in cases of functional limitations of the spine, such as scoliosis or orthopedic traumatic injuries [38,39,49,50,51,52,53].

In accordance with Vojta’s principles, the fundamental tenet of reflex locomotion involves the initiation of isometric contractions in postural muscles during stimulation. Prolonged and consistent stimulation exerts its influence on the musculature, joints, tendons, and ligaments. Additionally, Vojta’s reflex locomotion is intricately linked with both exteroceptors and interoceptors, serving as a source of afferent stimulation that enters the central nervous system [54]. The complete understanding of the mechanisms or neurobiological foundation behind the observed effects of Vojta therapy remains elusive. Vojta proposed the existence of a phylogenetically ancient “locomotion center” responsible for coordinating individual responses located beneath the upper brainstem. Supported by randomized controlled trials utilizing functional magnetic resonance imaging (MRI), there is speculation that the pontomedullary reticular formation plays a pivotal role in Vojta therapy. It is suggested to be involved in locomotor control, implicated in anticipatory postural control prior to walking initiation, by integrating descending cortical influences and ascending spinoreticular inputs [48].

The rehabilitation program consisted of the application of Vojta therapy in all cases included in the study. Each session lasted 20 min, with a frequency of three times per week, conducted by a physiotherapist specialized and certified in Vojta therapy. On the remaining days, parents were instructed to apply the same procedures.

**Figure 2:**
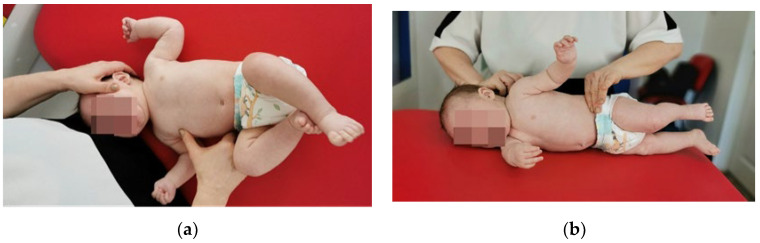
(**a**) Vojta therapy, reflex rolling, phase 1. (**b**) Vojta therapy, reflex rolling, phase 2.

1. Reflex rolling [55] (Figure 2a,b).

Phase 1: The infant is positioned in dorsal decubitus (DD), with the head turned towards the therapist and maintained in this position by the resistance given on the zygomatic bone, a position that stimulates the infant to turn the head in the opposite direction. This inhibition of head rotation movement induces a contraction of the scalene and SCM muscles opposite the stimulation area, while on the stimulated side, muscle stretching occurs. At the same time, the chest area (intercostal space 6–7) is stimulated. The stimulation duration is between 10 and 15 s, during which time kinesiological reactions are triggered, as follows: at the level of the upper limb on the occipital side (the scapula adheres to the rib cage, the scapulohumeral and elbow joint is flexed by 90°, and at the level of the fist in the medial position there is metacarpal abduction and an extension of the fingers); at the level of the upper limb on the facial side (the scapula adheres to the rib cage, a flexion, abduction, and external rotation occur in the scapulohumeral joint, flexion with supination is performed in the elbow joint, and the fist is positioned in dorsal extension and radial tilt, with metacarpals in abduction and fingers in extension); in the lower limb (in the hip and knee joint, a 90° angle is formed, the foot is positioned in the middle, the metatarsals are abducted, and the toes are in a median position). Stimulation is repeated 3 times on each side of the body.

Phase 2: The infant is positioned in lateral decubitus (LD) with the back turned towards the therapist, and two secondary areas are stimulated: the medial border of the scapula in the lower third and the anterior-superior iliac crest. Stimulation duration is between 10 and 15 s, 3 times on each side of the body until kinesiological reactions are triggered: in the upper limb on the supporting surface (the scapula adheres to the ribcage, in scapulohumeral joint a flexion of 90° and external rotation is produced, a slight flexion and pronation occurs in the elbow, and the fist is positioned in dorsal extension with radial tilt, with metacarpals in abduction and fingers in extension); in the upper limb which is not on the supporting surface (scapula—adheres to the thorax, in the scapulohumeral joint there is a flexion, abduction and external rotation, in the elbow there is a slight flexion with supination, and the fist is positioned in dorsal extension with radial inclination, with metacarpals in abduction and fingers in extension); in the lower limb on the support surface (in the coxofemoral joint there is a slight flexion with a tendency to extension in abduction and external rotation, the knee is maintained in slight flexion with a tendency to extension, and the foot is positioned medially with supination and inversion, with the metatarsals in abduction and toes in flexion); in the lower limb not on the support surface (in the coxofemoral joint there is a 90° flexion with abduction and external rotation, a 90° flexion in the knee, and the foot is positioned medially with abducted metatarsals and medial position of the toes).

**Figure 3:**
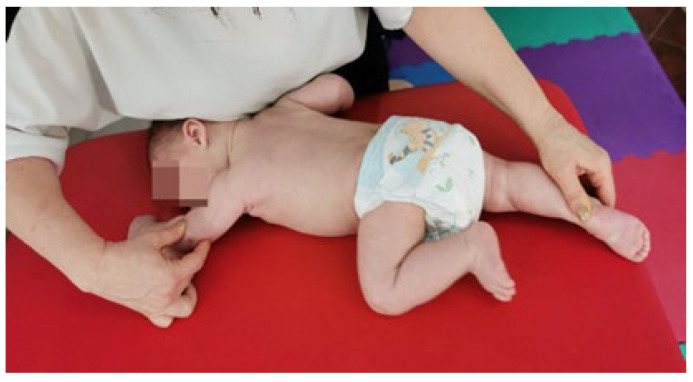
Vojta therapy, reflex crawling.

2. Reflex crawling [55] (Figure 3).

The infant is positioned in ventral decubitus (VD), the head is turned with the occipital side towards the therapist on the support surface, and the facial side towards the upper limb on the same side, the area of stimulation being the medial humeral epicondyle and the calcaneus area of the lower limb on the occipital side. From this position, the tendency of movement is to raise and turn the head towards the therapist (training the cervical muscles) while extending the arm on the occipital side and flexing the lower limb on the facial side (crossed movement). The abovementioned tendency to move is curbed, which causes a contraction and stretching of the cervical muscles. The stimulation is between 10 and 15 s, by changing the position of the body, stimulating each side 3 times, and watching for the triggering of kinesiological reactions: upper limb on the facial side (scapula adheres to the thorax, the elbow performs an extension, adduction, and external rotation with a 45° flexion with pronation, and the fist is positioned in dorsal extension with radial tilt, with metacarpals in abduction and fingers in flexion); upper limb on the occipital side (scapula—adheres to the thorax, the scapulohumeral joint is in flexion, abduction, and external rotation, the elbow in a 45° flexion with supination, and the fist in dorsal extension with radial tilt, with metacarpals in abduction and fingers in extension); lower limb on the occipital side (the hip joint is rotated at an angle of 45°, the foot is positioned medially at an angle of 90° with supination, with the metatarsals abducted and toes in flexion).

### 2.2. Statistical Analysis

For the statistical processing of study data, IBM SPSS Statistics for Windows, Version 22.0 IBM Corp was used. Nominal data were presented as absolute frequency and percentage, and continuous variables were expressed as average value and standard deviation. To compare means based on the numeric variables in the study, the independent samples t-test was employed. For comparing three or more group means where participants are the same in each group, the ANOVA test was utilized. Following the ANOVA procedure, to indicate the extent of the difference between the means of groups taken two by two, the Bonferroni post hoc test was applied. A significance level with a *p*-value < 0.05 was considered statistically significant.

## 3. Results

### Descriptive Statistical Analysis of Sample Characteristics

Of the 41 infants with confirmed diagnosis, 26 (63.4%) were boys and 15 (36.6%) were girls. The ratio between acquired and congenital forms of torticollis was 27 (65.9%) to 14 (34.1%). As for the affected side, 33 (80.5%) of infants had torticollis on the right side, while 8 (19.5%) had torticollis on the left side. Regarding the type of birth, 31 (75.6%) babies were born vaginally, while 10 (24.4%) came into the world by cesarean section. Regarding the intrauterine position, 9 (22.0%) infants were in pelvic presentation, while 32 (78.0%) were in cranial presentation. During the clinical examination for the identification of other changes due to sternocleidomastoid muscle contracture (SCM), deformities of the structure of the skull bones were observed, as follows: 16 (39.0%) with plagiocephaly and 25 (61.0%) with brachycephaly, in combination with facial asymmetries in 14 (34.1%) patients or body asymmetries in 31 (75.6%) patients (Table 1). These changes were caused by factors during the intrauterine period or by mechanisms during or after birth.

The average birth weight was 3500.98 g.

In Table 2, the average values of lateral flexions and rotations at all nine measuring moments can be observed. The average birth weight was 3500.98 g. The average recovery time of patients was 10.24 weeks, with a standard deviation of 3.52 weeks.

In Table 3, it was explored how sample characteristics differ according to types of torticollis. Among patients with congenital torticollis, 8 (57.1%) were male and 6 (42.9%) were female. Of those with acquired torticollis, 18 (66.7%) were male, and 9 (33.3%) were female. No significant differences were found between the two types of torticollis in terms of the sex of patients. Regarding the age of patients, higher shares of congenital torticollis were found in the first 3 months of life, compared to acquired torticollis, which is more common between the ages of 4 and 6 months. The result of the statistical analysis shows a statistically significant association (*p* = 0.007) between the type of torticollis and the age of patients. There is also a statistically significant association between pregnancy type and torticollis type (*p* = 0.003). In twin pregnancies, congenital muscle torticollis occurred more often than acquired. Congenital muscular torticollis occurred more frequently in cesarean sections (64.3%), while acquired torticollis occurred more frequently in natural births (96.3%), the association between birth type and torticollis type being statistically significant (*p* < 0.001). Also, congenital muscular torticollis occurred more frequently in pelvic presentations (64.3%), while acquired torticollis occurred more frequently in cranial presentations (100%). Plagiocephaly was found to be associated more frequently with congenital muscle torticollis, while brachycephaly was more commonly associated with acquired muscular torticollis. A significant association was also found between the presence of facial asymmetries and the type of torticollis. Facial asymmetries were more common (85.7%) in congenital muscle torticollis (*p* < 0.001). There are no statistically significant associations between torticollis type, on the one hand, and body asymmetry and affected side, on the other.

Birth weight did not differ significantly between patients with the two types of torticollis. The average birth weight of patients suffering from congenital torticollis was 3434.29 g, while the average birth weight of patients suffering from acquired torticollis was 3535.56 g (Table 4).

In the first three moments of measurement (initially, at 2 weeks and 4 weeks) there were significant differences in lateral flexion, which was significantly higher in those with congenital torticollis. In the following measurements, there were no statistical differences between the two types of torticollis regarding lateral flexion. In the case of rotation, too, statistically significant differences in averages were found between the two types of torticollis in the first three measurements, the number of degrees being higher in patients with acquired torticollis (Table 4).

The characteristics of the sample according to patient age are presented in Table 5. It is noted that congenital torticollis was more common at younger ages (1, 2, and 3 months), while acquired torticollis was more common at the ages of 4, 5, and 6 months. Plagiocephaly was also more common in the first months of life (1, 2, and 3 months), while brachycephaly was more common at the older ages (4, 5, and 6 months).

Recovery time was found to be significantly different depending on the age at which the patient was diagnosed (*p* < 0.001). As age progressed, the recovery period gradually increased from an average of 7.60 weeks in 1-month-old patients to an average of 14.67 weeks in 6-month-old patients. The weight of the infants did not influence the recovery period, as there was no statistically significant correlation between the parameters (Table 6).

**Figure 4:**
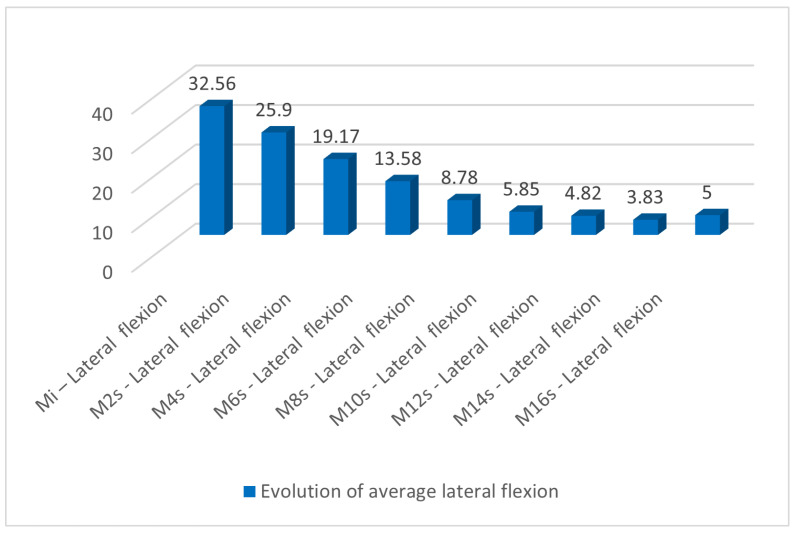
Evolution of average lateral flexion during the measurements.

In Table 7, the evolution of lateral flexion can be observed during the nine measurements. A permanent decrease in degrees of lateral flexion is observed from an average of 32.56 at the initial measurement to an average of 3.83 at week 16. This can also be seen in Figure 4.

**Figure 5:**
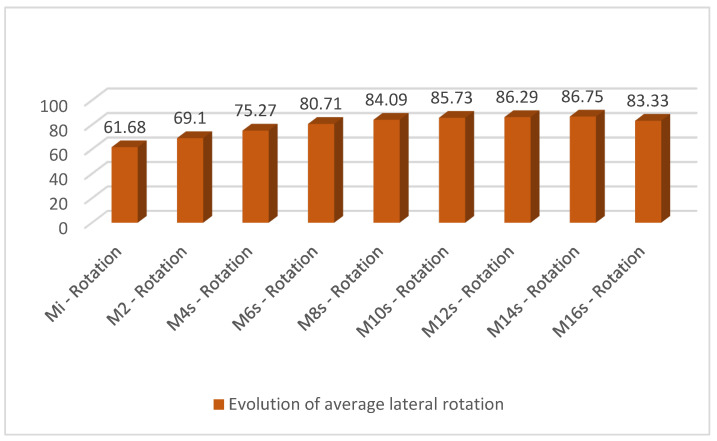
Evolution of average lateral rotation during the measurements.

In the case of rotation, the trend was continuously increasing from an average of 61.680 at the initial measurement to an average of 83.330 at week 16 (Table 8). This can also be seen in Figure 5.

In this study, the recovery time was analyzed according to a number of factors, such as gender, type of pregnancy, type of birth, type of presentation, cranial deformities, presence of facial asymmetry, presence of body asymmetry, type of torticollis, and affected part. Of all these factors, only the presence of body asymmetry proved to be significant in the evolution of recovery time. The presence of body asymmetry resulted in a significantly longer duration (11.16 weeks on average) of recovery time compared to patients who had no body asymmetries, for whom the average recovery time was 7.40 weeks. For the other factors, no significant differences in recovery time were found (Table 9).

Also, the recovery time was analyzed according to the age of the patients. The differences are statistically significant (*p* < 0.001), as seen in Table 10.

In Table 11, the results of the post hoc Bonferroni test are presented, which show the extent to which the averages of the groups taken two by two differ. Significant differences in recovery time were observed in young patients (1, 2, 3, and 4 months), where it had lower values compared to patients aged 5 and 6 months, for whom recovery times had higher average values.

**Figure 6:**
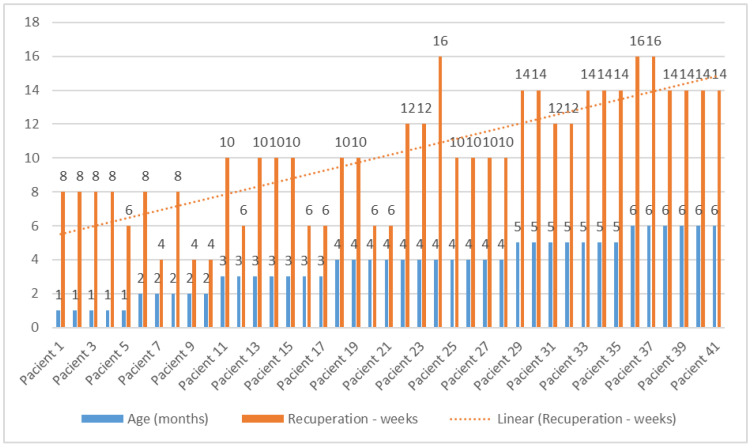
Rehabilitation duration based on patient age.

Figure 6 illustrates the recovery dynamics of infants based on the age at which therapy began, showing an upward trend in terms of the duration of recovery time starting from the age of 5 months.

## 4. Discussion

In this study, 41% of those who started therapy in the first 3 months of life were fully recovered after 4–6 weeks of therapy, and 35% after 8 weeks. However, 24% required therapy up to 10 weeks, all in the group of those who initiated therapy at 3 months of age. A total of 27% of the 41 infants in therapy were initiated at 4 months, and out of these, 55% with postural correction at 10 weeks, all with AMT, with only 27% requiring therapy between 12 and 16 weeks. In infants who started therapy at 5 and 6 months of age, representing 32% of the total group, difficulties were encountered in obtaining complete results, especially among those aged 6 months, with 15% of those with CMT seeing no improvements in measurements from 14 to 16 weeks of age where the use of a cranial orthosis was recommended, and 23% experiencing a plateau in measurements from 10 to 14 weeks, requiring the use of a cervical collar in conjunction with therapy.

Out of the total number of patients (n = 41), 5 infants who commenced therapy at 6 months did not achieve complete rehabilitation after the implementation of the physical therapy treatment over a duration of 16 weeks, while 36 infants were considered fully recovered.

Most infants with torticollis included in the study began therapy at the age of 4 months. They were not referred to a physical therapist before that, which required their inclusion in a longer recovery program. The duration of recovery was influenced by the age at which therapy was initiated and the presence of body asymmetries.

Thus, patients with muscular torticollis aged one month required on average 8 weeks for a complete recovery, while those aged 4–6 months required 16 weeks (Figure 6). It was found that a higher number of male infants were diagnosed with torticollis, a finding that is also evident in other studies described in the databases (63.41% vs. 36.59%) [26,56,57].

The interpretation of the statistical data does not highlight a direct association between the type of torticollis and body asymmetries. However, in the specialized literature, torticollis is recognized as a possible cause of body asymmetries. A retrospective study conducted on a sample of 130 children with congenital muscular torticollis showed that 39.22% of them developed pelvic misalignment syndrome over time, with or without compensatory scoliosis [9], a conclusion also reached by Wilczyński J. in his study [58].

Early kinetotherapeutic intervention in the case of torticollis, regardless of its nature, whether congenital or acquired, has had a positive impact on the integrity of muscular and articular structures, supporting the progress of motor development within normal limits [59,60]. According to specialized literature, Vojta therapy can have a beneficial impact on individuals with cerebral palsy [41,46,61,62], scoliosis [63,64], body asymmetries [65], trunk control and postural balance in children with central hypotonia [66], and hip dislocation [60].

The initiation of early therapy for infant torticollis may be attributed to the increased plasticity of muscles and joints during early developmental stages, heightened plasticity of the developing nervous system, and more effective parental engagement in the therapeutic process. This brings significant benefits such as rapid recovery, prevention of complications, optimal development of the musculoskeletal system, and reduced need for invasive interventions.

The presence of asymmetries resulting from torticollis can lead to musculoskeletal imbalances and the development of compensatory mechanisms, necessitating a more comprehensive and prolonged therapy for restoring normal alignment. Severe asymmetries may impact the infant’s functional activities, requiring focused and extended therapeutic interventions, especially in older infants where asymmetries may be more established and challenging to correct within a shorter timeframe.

Strictly regarding congenital torticollis in the study conducted by Ah Young J [67] on a group of 118 cases with CMT who received physical therapy, it was demonstrated that the recovery process is influenced by three factors: sternocleidomastoid muscle thickness, low birth weight, and fetal position. Thus, children born with pelvic presentation, a low birth weight, or with a more developed sternocleidomastoid muscle require longer rehabilitation treatment. However, despite these variables with a less favorable outlook, it was found that factors related to the timing of the initiation of physiotherapy recovery, i.e., early intervention, can positively influence prognosis and can achieve comparable outcomes to those of individuals not in the risk group. This allows for the prevention of complications, an aspect that the study highlighted.

In most cases, conservative treatments lead to positive results; however, surgery may be considered optimal for patients in whom a continuous degradation of cervical range of motion is observed, or for those in whom the maxillofacial deformity evolves progressively. Even though this method is not a focal point of the research, it is important to remember that studies show that facial asymmetry in patients with torticollis can be partially improved if surgical release is performed before the age of 10 years [30,68].

Kinesiotherapy is a real support and a primary way of treating musculoskeletal deformities.

Taking into account the data from the study showing that sternocleidomastoid muscle contracture is frequently accompanied by skull deformities, in 39.02% for brachycephalies and 65.85% for plagiocephalies, it is essential to stress the importance of prompt referral of patients to a physiotherapist experienced in the treatment of congenital muscular torticollis. This action may help avoid more costly or invasive interventions, such as the use of cranial orthoses or surgical procedures [35]. In their study, Pascal P [69] pointed out, following the application of conservative techniques for rehabilitation in congenital torticollis, that maximum effectiveness and complete recovery of over 50% of infants was achieved when therapy was initiated before the age of 3 months and applied at a frequency of three times per week in parallel with rehabilitation sessions at home. The results of our study are consistent with theirs. A total of 68% were infants referred for therapy up to and including 4 months of age, with 89% of them having made a complete recovery within 10 weeks after the therapy was started.

Also, in a study conducted on the superiority of conservative treatments for plagiocephaly and congenital torticollis, it was found that repositioning, including the duration of this procedure, and stretching exercises performed by a specialist were low-risk but potentially significant interventions. These therapeutic options can be considered an effective and economical alternative for parents [25].

In other studies, Bobath and Vojta’s techniques have been approached as recovery therapies. An especially pertinent conclusion was reached by Jung MW [65] who, in a study conducted in 2017 on a group of 65 infants with MCT, sought to highlight the best results obtained in the shortest possible time by applying different therapeutic methods to different groups, namely Bobath on one group, and Vojta on the other group. After 8 weeks, there were significantly better results in the group receiving Vojta therapy, thus confirming the effectiveness of this approach, which corroborates our findings.

In a systematic review of clinical studies focusing on infants under 18 months with congenital muscular torticollis published between 1990 and 2018, and treated with stretching therapy, it was concluded that this therapy is effective when applied early (before the age of 3 months in infants). Factors influencing the success of the treatment included parental adherence and the addition of a home exercise program [69].

The clinical measurements in the study involved the use of a goniometer, although there are other instruments available to assess joint range of motion (ROM). The use of these alternative tools is proving to be problematic in infants because of the low level of cooperation during physical assessments. In another study, in addition to the goniometer, photography and three-dimensional (3D) scanning techniques were used; their results showed significant efficacy with the 3D scanning method of the deviation angle, the advantage of these tools being greater in older children [70].

The recognition of the importance of a differential diagnosis is essential to distinguish between severe infections or inflammation in the neck area, which can cause discomfort and muscle stiffness [71]. Also, torticollis can be, in certain situations, the sole symptom in almost 39% of cases of nervous system tumors [72].

Although Vojta therapy is recognized as effective, according to research, the implementation of this therapy influences the emotional state of parents, given that the child exhibits crying during the activation of reflexes [73]. Therefore, according to Kiebzak’s study [74], it is observed that stimulation through Vojta therapy leads to temporary increases in cortisol levels (the stress hormone) in infants with central coordination disorder; however, this level subsequently decreases, reaching normal values. In a comparative study where two therapeutic techniques, Bobath and Vojta, were applied, it was concluded that the emotional impact on parents was more pronounced in the Vojta therapy group [75].

Future investigations will involve a more in-depth analysis of predictions related to torticollis in children through formalization. This approach aims to enhance public understanding of the research process. The formal model will facilitate the identification of all research stages, ultimately making the dissemination of findings more accessible [76,77,78].

The Vojta therapy should not be used in acute inflammations, acute febrile states, vaccinations with live viruses (10 days after vaccination), or in certain conditions such as heart diseases. In some situations, the previously mentioned contraindications may lead to the discontinuation of therapy sessions with a potential regression, as there is no continuity in treatment [79].

This study underscores its nature as a retrospective pilot study conducted at a single center, involving analysis of a modest cohort. To enhance the robustness and applicability of the findings, a broader, more diverse sample from various centers is recommended. Such an approach could offer a more representative snapshot of the population. Furthermore, the study’s limited duration may not capture the enduring effects or relapse rates in infants, necessitating longitudinal follow-up for a comprehensive grasp of the therapy’s impact. The absence of a control group undergoing different or no treatment poses a challenge in attributing observed improvements solely to Vojta therapy.

While acknowledging these constraints, this study contributes valuable insights into infantile torticollis. Future research endeavors should address the aforementioned limitations to advance our understanding of this condition.

Additionally, there is a need for further research, such as randomized trials comparing the outcomes of early versus late initiation of therapy.

## 5. Conclusions

The findings from this study suggest that there may be a correlation between early initiation of therapy and favorable outcomes in the recovery process. Infants who commenced therapy within the first 3 months demonstrated higher rates of complete recovery within a 4–6-week period. However, caution is advised in drawing definitive conclusions about efficacy due to the study’s observational design. Conversely, those who initiated therapy later, particularly after 4 months of age, encountered challenges in achieving complete recovery. It is worth noting that early intervention appeared to positively impact motor development and mitigate compensatory skeletal changes. The primary factors influencing the duration of recovery were identified as the presence of body asymmetries and the age at which therapy was initiated.

## Figures and Tables

**Table 1:** Main characteristics of the studied sample.

		Frequency	Percent
Gender	Male	26	63.4
	Female	15	36.6
Age (months)	1	5	12.2
	2	5	12.2
	3	7	17.1
	4	11	26.8
	5	7	17.1
	6	6	14.6
Pregnancy	Single	37	90.2
	Twin	4	9.8
Birth	Natural	31	75.6
	Cesarean section	10	24.4
Presentation	Cranial	32	78.0
	Pelvic	9	22.0
Cranial deformities	Plagiocephaly	16	39.0
	Brachycephaly	25	61.0
Facial asymmetry	Yes	14	34.1
	No	27	65.9
Body asymmetry	Yes	31	75.6
	No	10	24.4
Torticollis (type)	Congenital muscle	14	34.1
	Acquired muscle	27	65.9
Affected side	Left	8	19.5
	Right	33	80.5

**Table 2:** Average values and standard deviations of the characteristics of the studied sample.

	N = 41		Average	Standard Deviation
	Validated Data	Missing Data		
Birth weight (g)	41	0	3500.98	503.104
Mi-Lateral flexion	41	0	32.56	9.503
Mi-Rotation	41	0	61.68	6.486
M2s-Lateral Flexion	41	0	25.90	8.694
M2s-Rotation	41	0	69.10	7.228
M4s-Lateral Flexion	41	0	19.17	8.820
M4s-Rotation	41	0	75.27	7.804
M6s-Lateral Flexion	38	3	13.58	7.258
M6s-Rotation	38	3	80.71	6.328
M8s-Lateral Flexion	32	9	8.78	5.813
M8s-Rotation	32	9	84.09	4.973
M10s-Lateral Flexion	26	15	5.85	5.446
M10s-Rotation	26	15	85.73	4.313
M12s-Lateral Flexion	17	24	4.82	3.893
M12s-Rotation	17	24	86.29	3.331
M14s-Lateral Flexion	12	29	3.83	4.366
M14s-Rotation	12	29	86.75	4.070
M16s-Lateral Flexion	3	38	5.00	5.000
M16s-Rotation	3	38	83.33	5.774
Recovery time (weeks)	41	0	10.24	3.527

**Table 3:** Comparative analysis of all sample characteristics according to the types of torticollis.

Parameters		Congenital Muscular Torticollis		Acquired Muscular Torticollis		*p*
		Frequency	Percent	Frequency	Percent	
Gender	Male	8	57.1	18	66.7	0.548
	Female	6	42.9	9	33.3	
Age (months)	1	4	28.6	1	3.7	0.007
	2	2	14.3	3	11.1	
	3	5	35.7	2	7.4	
	4	1	7.1	10	37.0	
	5	0	0.0	7	25.9	
	6	2	14.3	4	14.8	
Pregnancy	Single	10	71.4	27	100.0	0.003
	Twin	4	28.6	0	0.0	
Birth	Natural	5	35.7	26	96.3	<0.001
	Cesarean section	9	64.3	1	3.7	
Presentation	Cranial	5	35.7	27	100.0	<0.001
	Pelvic	9	64.3	0	0.0	
Cranial deformities	Plagiocephaly	14	100.0	2	7.4	<0.001
	Brachycephaly	0	0.0	25	92.6	
Facial asymmetry	Yes	12	85.7	2	7.4	<0.001
	No	2	14.3	25	92.6	
Body asymmetry	Yes	12	85.7	19	70.4	0.278
	No	2	14.3	8	29.6	
Affected side	Left	4	28.6	4	14.8	0.292
	Right	10	71.4	23	85.2	

**Table 4:** Analysis of birth weight and the type of torticollis.

	Torticollis (Type)	N = 41	Average	Std. Deviation	*p*-Value
Birth weight (g)	Congenital muscle	14	3434.29	751.099	0.548
	Acquired muscle	27	3535.56	322.017	
Mi-Lateral Flexion	Congenital muscle	14	40.71	5.717	<0.001
	Acquired muscle	27	28.33	8.260	
M2s-Lateral Flexion	Congenital muscle	14	33.21	4.791	<0.001
	Acquired muscle	27	22.11	7.817	
M4s-Lateral Flexion	Congenital muscle	14	24.86	5.142	0.002
	Acquired muscle	27	16.22	8.946	
M6s-Lateral Flexion	Congenital muscle	14	15.43	6.370	0.235
	Acquired muscle	24	12.50	7.650	
M8s-Lateral Flexion	Congenital muscle	13	6.69	7.250	0.093
	Acquired muscle	19	10.21	4.224	
M10s-Lateral Flexion	Congenital muscle	7	6.71	6.993	0.632
	Acquired muscle	19	5.53	4.948	
M12s-Lateral Flexion	Congenital muscle	4	6.50	5.066	0.341
	Acquired muscle	13	4.31	3.545	
M14s-Lateral Flexion	Congenital muscle	3	6.33	3.215	0.272
	Acquired muscle	9	3.00	4.528	
M16s-Lateral Flexion	Congenital muscle	3	5.00	5.000	-
	Acquired muscle	0 ^a^	-	-	
Mi-Rotation	Congenital muscle	14	56.07	5.313	<0.001
	Acquired muscle	27	64.59	4.987	
M2-Rotation	Congenital muscle	14	63.21	5.236	<0.001
	Acquired muscle	27	72.15	6.194	
M4s-Rotation	Congenital muscle	14	70.50	6.560	0.004
	Acquired muscle	27	77.74	7.320	
M6s-Rotation	Congenital muscle	14	79.29	6.450	0.295
	Acquired muscle	24	81.54	6.241	
M8s-Rotation	Congenital muscle	13	85.23	6.044	0.292
	Acquired muscle	19	83.32	4.083	
M10s-Rotation	Congenital muscle	7	85.14	5.786	0.682
	Acquired muscle	19	85.95	3.808	
M12s-Rotation	Congenital muscle	4	83.75	4.787	0.080
	Acquired muscle	13	87.08	2.499	
M14s-Rotation	Congenital muscle	3	82.67	4.619	0.037
	Acquired muscle	9	88.11	3.018	
M16s-Rotation	Congenital muscle	3	83.33	5.774	-
	Acquired muscle	0 ^a^	-	-	

^a^ Cannot be computed because at least one of the groups is empty.

**Table 5:** Comparative analysis of sample characteristics by age.

		Age (Months)						*p*-Value
		1	2	3	4	5	6	
Gender	Male	3 (60.0%)	2 (40.0%)	4 (57.1%)	7 (63.0%)	4 (57.1%)	6 (100.0%)	0.427
	Female	2 (40.0%)	3 (60.0%)	3 (42.9%)	4 (36.4%)	3 (42.9%)	0 (0.0%)	
Pregnancy	Single	4 (80.0%)	4 (80.0%)	5 (71.4%)	11 (100%)	7 (100%)	6 (100%)	0.252
	Twin	1 (20.0%)	1 (20.0%)	2 (28.6%)	0 (0.0%)	0 (0.0%)	0 (0.0%)	
Birth	Natural	3 (60.0%)	3 (60.0%)	3 (42.9%)	11 (100%)	7 (100%0	4 (66.7%)	0.043
	Cesarean section	2 (40.0%)	2 (40.0%)	4 (57.1%)	0 (0.0%)	0 (0.0%)	2 (33.3%)	
Presentation	Cranial	3 (60.0%)	3 (60.0%)	4 (57.1%)	11 (100%)	7 (100%)	4 (66.7%)	0.101
	Pelvic	2 (40.0%)	2 (40.0%)	3 (42.9%)	0 (0.0%)	0 (0.0%)	2 (33.3%)	
Cranial deformities	Plagiocephaly	4 (80.0%)	2 (40.0%)	5 (71.4%)	3 (27.3%)	0 (0.0%)	2 (33.3%)	0.037
	Brachycephaly	1 (20.0%)	3 (60.0%)	2 (28.6%)	8 (72.7%)	7 (100%)	4 (66.7%)	
Facial asymmetry	Yes	4 (80.0%)	2 (40.0%)	3 (42.9%)	3 (27.3%)	0 (0.0%)	2 (33.3%)	0.115
	No	1 (20.0%)	3 (60.0%)	4 (57.1%)	8 (72.7%)	7 (100%)	4 (66.7%)	
Body asymmetry	Yes	2 (40.0%)	2 (40.0%)	6 (85.7%)	8 (72.7%)	7 (100%)	6 (100%)	0.042
	No	3 (60.0%)	3 (60.0%)	1 (14.3%)	3 (27.3%)	0 (0.0%)	0 (0.0%)	
Torticollis (type)	Congenital muscle	4 (80.0%)	2 (40.0%)	5 (71.4%)	1 (9.1%)	0 (0.0%)	2 (33.3%)	0.007
	Acquired muscle	1 (20.0%)	3 (60.0%)	2 (28.6%)	10 (90.9%)	7 (100%)	4 (66.7%)	
Affected side	Left	2 (40.0%)	0 (0.0%)	2 (28.6%)	3 (27.3%)	1 (14.3%)	0 (0.0%)	0.427
	Right	3 (60.0%)	5 (100%)	5 (71.4%)	8 (72.7%)	6 (85.7%)	6 (100%)	

**Table 6:** Analysis of recovery duration in relation to age and weight.

Age (Months)		N = 41	Average	Std. Deviation	*p*-Value
Birth weight (g)	1	5	3380.00	779.102	0.824
	2	5	3292.00	676.254	
	3	7	3454.29	680.658	
	4	11	3550.91	328.860	
	5	7	3690.00	150.222	
	6	6	3518.33	506.850	
Recovery period (weeks)	1	5	7.60	0.894	<0.001
	2	5	5.60	2.191	
	3	7	8.57	2.507	
	4	11	10.18	2.750	
	5	7	13.43	0.976	
	6	6	14.67	1.033	

**Table 7:** Evolution of lateral flexion.

		Mi–Lateral Flexion	M2s-Lateral Flexion	M4s-Lateral Flexion	M6s-Lateral Flexion	M8s-Lateral Flexion	M10s-Lateral Flexion	M12s-Lateral Flexion	M14s-Lateral Flexion	M16s-Lateral Flexion
N	Valid	41	41	41	38	32	26	17	12	3
	Missing	0	0	0	3	9	15	24	29	38
Average		32.56	25.90	19.17	13.58	8.78	5.85	4.82	3.83	5.00

**Table 8:** Evolution of lateral rotation.

		Mi-Rotation	M2-Rotation	M4s-Rotation	M6s-Rotation	M8s-Rotation	M10s-Rotation	M12s-Rotation	M14s-Rotation	M16s-Rotation
N	Valid	41	41	41	38	32	26	17	12	3
	Missing	0	0	0	3	9	15	24	29	38
Average		61.68	69.10	75.27	80.71	84.09	85.73	86.29	86.75	83.33

**Table 9:** Analysis of recovery time in relation to the main characteristics.

	Gender	N = 41	Average	Std. Deviation	*p*-Value
Sex	Male	26	10.62	3.383	0.381
	Female	15	9.60	3.795	
Pregnancy	Single	37	10.43	3.594	0.304
	Twin	4	8.50	2.517	
Birth	Natural	31	10.39	3.518	0.653
	Cesarean section	10	9.80	3.706	
Presentation	Cranial	32	10.25	3.547	0.984
	Pelvic	9	10.22	3.667	
Cranial deformities	Plagiocephaly	16	10.50	3.225	0.715
	Brachycephaly	25	10.08	3.763	
Facial asymmetry	Yes	14	10.86	3.207	0.430
	No	27	9.93	3.700	
Body asymmetry	Yes	31	11.16	3.174	0.002
	No	10	7.40	3.134	
Torticollis (type)	Congenital muscle	14	10.29	3.407	0.957
	Acquired muscle	27	10.22	3.651	
Affected side	Left	8	10.25	3.615	0.996
	Right	33	10.24	3.562	

**Table 10:** Recovery time (weeks).

Age	N = 41	Average	Std. Deviation	*p*-Value
1	5	7.60	0.894	<0.001
2	5	5.60	2.191	
3	7	8.57	2.507	
4	11	10.18	2.750	
5	7	13.43	0.976	
6	6	14.67	1.033	

**Table 11:** Multiple comparisons dependent variable: recovery time (weeks) Bonferroni.

(I) Age (Months)	(J) Age (Month)	Average Difference (I − J)	*p*-Value
1	2	2.000	1.000
	3	−0.971	1.000
	4	−2.582	0.379
	5	−5.829 *	0.000
	6	−7.067 *	0.000
2	1	−2.000	1.000
	3	−2.971	0.273
	4	−4.582 *	0.003
	5	−7.829 *	0.000
	6	−9.067 *	0.000
3	1	0.971	1.000
	2	2.971	0.273
	4	−1.610	1.000
	5	−4.857 *	0.001
	6	−6.095 *	0.000
4	1	2.582	0.379
	2	4.582 *	0.003
	3	1.610	1.000
	5	−3.247 *	0.035
	6	−4.485 *	0.002
5	1	5.829 *	0.000
	2	7.829 *	0.000
	3	4.857 *	0.001
	4	3.247 *	0.035
	6	−1.238	1.000
6	1	7.067 *	0.000
	2	9.067 *	0.000
	3	6.095 *	0.000
	4	4.485 *	0.002
	5	1.238	1.000

* The mean difference is significant at the 0.05 level.

## Data Availability

The data presented in this study are available on request from the corresponding author.
